# Treatment of Sub-Involution of the Uterus

**Published:** 1880-08

**Authors:** James Braithwaite

**Affiliations:** London, Lecturer on Diseases of Women and Children, Leeds School of Medicine; and Surgeon to the Hospital for Women and Children


### NOTES ON THE TREATMENT OF SUB-INVOLUTION OF THE UTERUS.

BY JAMES BRAITHWAITE, M. D., LONDON,

Lecturer on Diseases of Women and Children, Leeds School of Medicine; and Surgeon to the Hospital for Women and Children.

THE writer having long felt the want of a perfectly safe and at the same time efficient means of treating uncomplicated subinvolution of the uterus, gladly tried for the first time, about two years ago, a plan shown him by Dr. Wynn Williams, at the Samaritan Hospital. This treatment is in common use by Dr. Williams amongst the out-patients of that hospital, and in the hands of the writer has been found to deserve all the confidence felt in it by its original author, with whose permission this note, which was at first only intended for a local medical society, is now published by request.

It may be premised that this treatment is less suitable in cases complicated by endometritis, which should be treated previously for that disease.

The treatment consists in the application to the interior of the uterine cavity of a solution composed of equal parts of iodine, iodide of potassium, and spirits of wine. The first two ingredients dissolve readily in the third.

The patient being in the dorsal position, a Fergusson's speculum inserted, and all mucus wiped away, it is a good plan to pass a sound through the cervical canal, as the iodine application is more readily passed afterwards. The sound used for this purpose should be curved only in its terminal half inch, or a trifle more, and this curve should be of about forty-five degrees angle, in order to be easily used through a speculum. A small whalebone bougie, having its slightly bulbous extremity removed, or any long and finely tapering piece of whalebone, is to be wrapped round with cotton wool in its terminal four inches, or to a greater length than that of the enlarged uterine cavity. About two inches of this is saturated with the iodine solution, and is rapidly passed up to the fundus, and there allowed to remain for a few moments. To facilitate the rapid insertion of the whalebone, it should be slightly curved towards its extremity, and sufficiently fine to allow of its bending very readily. This is withdrawn in about a minute, the cotton of course coming away upon the whalebone, and being then stripped from it. The effect of the application of this powerful solution of iodine is to cause immediate forcible contraction of the uterine muscular fibre, as may be inferred from the difficulty of passing the cotton a second time soon after its withdrawal. This contraction, however, only occurs when the contractility of the muscular fibre is not lowered by inflammation. As, however, a second application should not be necessary, the contraction passes unnoticed. This solution should be applied twice between the periods, and continued according to the results. The lessening of the size of the uterus is generally marked and rapid, so that on introducing a sound at the end of a week after the first application, a uterus previously measuring three and a half inches may be expected to have lost half an inch of its internal length. Seldom more than three or four applications have been made; and cases chosen which were uncomplicated with much tenderness of the uterus, and unattended with the dirty brownish discharge of endometritis. It is in the experience of the writer a mistake to mix up such cases with sub-involution, and treat them with one remedy, as advised by a late writer upon the use of "iodized phenol." Treat the endometritis first, either with ordinary tincture of iodine, as recommended by Dr. Tilt, and as was the habit of the late Dr. Tyler Smith, or with carbolic acid after Dr. Playfair's plan, and then, if the uterus does not lessen in size as much as desired, use this strong solution of iodine. If this is reversed, the strong iodine will be found to aggravate the inflammation and the carbolic acid inefficient in curing the sub-involution. The only plan of treatment which seems equally efficient with the one described, is that used by Dr. Atthill, of Dublin, the introduction into the uterus of ten grains of solid nitrate of silver. This treatment appears rather heroic, and at this side of the Irish Channel would probably set up unpleasant inflammatory action.

In conclusion, this short article only expresses the experience of its writer, who claims no merit whatever, and who takes this opportunity of thanking the original author of the treatment for a valuable hint in practice, which has helped him over a number of cases which he would otherwise have found it difficult to treat rapidly and successfully, and without fear of doing harm by setting up inflammation or obliterating the uterine cavity by producing adhesion of its surfaces.—*Obst. Journal of Great Britain and Ireland.*

